# Evaluation of the Antioxidant Activity of Cell Extracts from Microalgae

**DOI:** 10.3390/md11041256

**Published:** 2013-04-17

**Authors:** A. Catarina Guedes, Maria S. Gião, Rui Seabra, A. C. Silva Ferreira, Paula Tamagnini, Pedro Moradas-Ferreira, F. Xavier Malcata

**Affiliations:** 1 CBQF/Biotechnology College, Catholic University of Portugal, Rua Dr. António Bernardino de Almeida, Porto P-4200-072, Portugal; E-Mails: acatarinaguedes@gmail.com (A.C.G.); msilvagiao@hotmail.com (M.S.G.); acferreira@esb.ucp.pt (A.C.S.F.); 2 CIMAR/CIIMAR—Interdisciplinary Centre of Marine and Environmental Research, University of Porto, Rua dos Bragas nº 177, Porto P-4050-123, Portugal; 3 IBMC—Institute for Molecular and Cell Biology, University of Porto, Rua do Campo Alegre nº 823, Porto P-4150-180, Portugal; E-Mails: ruisea@gmail.com (R.S.); pmtamagn@ibmc.up.pt (P.T.); pmferrei@ibmc.up.pt (P.M.-F.); 4 Department of Biology, Faculty of Sciences, University of Porto, Rua do Campo Alegre, Edifício FC4, Porto P-4169-007, Portugal; 5 ICBAS—Institute of Biomedical Sciences Abel Salazar, University of Porto, Largo Abel Salazar nº 2, Porto P-4099-003, Portugal; 6 Department of Chemical Engineering, University of Porto, Rua Dr. Roberto Frias, Porto P-4200-465, Portugal

**Keywords:** 16S rDNA, ABTS^•+^, deoxyribose, DNA, phage P22/*Salmonella*, Ames test

## Abstract

A growing market for novel antioxidants obtained from non-expensive sources justifies educated screening of microalgae for their potential antioxidant features. Characterization of the antioxidant profile of 18 species of cyanobacteria (prokaryotic microalgae) and 23 species of (eukaryotic) microalgae is accordingly reported in this paper. The total antioxidant capacity, accounted for by both water- and lipid-soluble antioxidants, was evaluated by the (radical cation) ABTS method. For complementary characterization of cell extracts, a deoxyribose assay was carried out, as well as a bacteriophage P22/*Salmonella*-mediated approach. The microalga *Scenedesmus obliquus* strain M2-1 exhibited the highest (*p* > 0.05) total antioxidant capacity (149 ± 47 AAU) of intracellular extracts. Its scavenger activity correlated well with its protective effects against DNA oxidative damage induced by copper(II)-ascorbic acid; and against decay in bacteriophage infection capacity induced by H_2_O_2_. Finally, performance of an Ames test revealed no mutagenic effects of the said extract.

## 1. Introduction

The last decade has witnessed a growing interest in compounds from natural sources that bear antioxidant properties because these compounds may provide relevant contributions to maintain health, e.g., via regular ingestion as part of formulated foods. Indeed evidence gathered in a large number of worldwide studies supports the role of antioxidants in the prevention of and in the growth control of certain tumours, as well as in the incidence and severity of cardiovascular and degenerative diseases [1,2,3]. Furthermore, a favourable effect upon human life span via delays in ageing has been claimed, based on a decrease in such biomarkers as protein carbonyls produced via oxidative damage. The growing market for novel antioxidants obtained from non-expensive, less conventional sources therefore justifies comprehensive searches encompassing unusual sources—as is the case with microalgae.

Microalgae are the basis of the food chain in aquatic ecosystems; with the aid of solar energy, they can use H_2_O and CO_2_ to synthesize complex organic compounds—and subsequently accumulate and/or secrete many primary and secondary metabolites of interest [4,5,6]. Furthermore, microalgae exhibit adaptative responses to oxidative stresses, via stimulation of their antioxidant defence system [7] that consists of both enzymatic and non-enzymatic mechanisms: superoxide dismutase, catalase, glutathione reductase and ascorbate peroxidase are key enzymes in the former, whereas the non-enzymatic counterpart includes such mediator compounds as ascorbic acid, reduced glutathione, tocopherols, carotenoids and phycocyanin [8].

The research effort described in this paper attempts to contribute to expand the uses of microalgae, namely in the food industry, by specifically taking advantage of their antioxidant features. Therefore, the experimental work encompassed screening of several unusual microalgae for antioxidant behaviour using complementary analytical approaches; a preliminary test of mutagenicity was also carried out, as required before any given microbial extract undergoes further in-depth characterization regarding high-added value functional compound(s). Both intra- and extra-cellular extracts were considered, so as to comprehensively characterize the strains in terms of total antioxidant capacity; the former are normally more abundant, but the latter would be more interesting from a processing point of view owing to easier recovery from the microbial broth. Screening started with generic assays for antioxidant features; only those strains bearing a good potential were subjected to more detailed assays, so as to reasonably comply with limited laboratory resources while maximizing experimental efficacy.

## 2. Results and Discussion

### 2.1. Microorganisms

The main objective of this research effort was to identify less conventional microalga cells harbouring compounds with antioxidant capacity. Hence, the set of microorganisms tested included unusual strains/species maintained in culture collections, as well as wild ones isolated from unique ecosystems in Portugal (see Table 1, Table 2).

Environmental isolates were identified via a polyphasic approach, using both morphological characteristics and partial sequencing of 16S rRNA; such isolates were tentatively named after their closest cultured relative.

**Table 1:** Total antioxidant capacity (average ± standard deviation) of cyanobacterial extracts, expressed as ABTS radical scavenging activity, in AAU ((mg/L_equivalent ascorbic acid_)/μg_chlorophyll *a*_).

Strain	Origin	Culture Medium	Antioxidant Power	
			Intracellular	Extracellular
**Unicellular**				
*Chlorococcus* *giganteus*	ACOI 768	BG11	2.95 ± 0.92	nd
		OHM	1.01 ± 0.19	nd
*Cyanothece* sp.	ATCC 51142	BG11	9.78 ± 0.73	nd
*Gloeobacter* *violaceus*	PCC 7421	BG11	38.10 ± 6.03 ^a,b,c^	0.07 ± 0.01 ^b^
*Gloeothece* sp.	ATCC 27152	BG11_0_	5.12 ± 1.09	0.03 ± 0.00
*Synechocystis* sp.	PCC 6803	BG11	2.97 ± 0.46	nd
*Synechocystis* *salina*	ACOI 48	BG11	7.98 ± 1.01	nd
**Filamentous**				
*Arthrospira* *platensis*	ATCC 29408	ASW-BG11	3.23 ± 0.59	nd
*Leptolyngbya* sp.	PCC 73110	BG11	2.68 ± 0.43	nd
*Leptolyngbya* sp.	PCC 7410	BG11	2.32 ± 0.31	nd
M2-7 (*Limnothrix* sp.) *	Aquaculture biofilters	BG11	17.00 ± 8.47 ^a,b,c,d^	0.01 ± 0.00 ^a,c^
*Lyngbya* *majuscula*	CCAP 1446/4	BG11	3.46 ± 1.04	nd
**Filamentous heterocystous**				
*Anabaena* *variabilis*	ATCC 29413	BG11_0_	2.78 ± 0.48	nd
		BG11	3.29 ± 0.89	nd
J52 (*Anabaena planctonica*) *	Maranhão lagoon	BG11	35.90 ± 6.99 ^a,b,c^	0.01 ± 0.00 ^b^
*Nodularia* *harveyana*	ACOI 729	BG11	1.84 ± 0.57	0.01 ± 0.00
*Nostoc* *carneum*	ACOI 650	BG11_0_	6.78 ± 1.33	nd
		BG11	5.00 ± 0.60	nd
*Nostoc* *muscorum*	CCAP 1453/12	BG11_0_	2.02 ± 0.46	nd
		BG11	3.06 ± 0.32	nd
*Nostoc* *punctiforme*	PCC 73102	BG11_0_	2.22 ± 0.36	0.07 ± 0.01
		BG11	9.42 ± 1.34	nd
*Scytonema* *obscurum*	ACOI 573	BG11	14.80 ± 1.76 ^c,d^	nd

* Wild strain, identified using morphological characteristics and partial sequencing of 16S rDNA. ^a–d^ Means within the same column, without a common superscript, are significantly different (*p* < 0.05). nd (not detected). ACOI—Coimbra Collection of Algae (University of Coimbra, Portugal); ATCC—American Type Culture Collection (USA); CBSC—Carolina Biological Supply Company (USA); CCAP—Culture Collection of Algae and Protozoa (UK); IPIMAR (Portugal); and PCC—Pasteur Culture Collection (France).

**Table 2:** Total antioxidant capacity (average ± standard deviation) of microalgal extracts, expressed as ABTS radical scavenging activity, in AAU ((mg/L_equivalent ascorbic acid_)/μg_chlorophyll *a*_).

Class/Species	Origin	Culture Medium	Antioxidant Power	
			Intracellular	Extracellular
**Bacillariophyceae**				
*Phaeodactilum* *tricornutum*	SERI-S/PHAEO-1 (TFX-1)	ASW	8.8 ± 1.84	nd
**Chlorophyceae**				
*Chlorella* *vulgaris*	CBSC	OHM	10.2 ± 1.93	nd
*C.* *vulgaris*	ACOI 879	OHM	7.48 ± 1.24	nd
*Haematococcus* *pluvialis*	CCAP 34/7	OHM	49.80 ± 10.10 ^g^	nd
*Scenedesmus* *maximus*	ACOI 318	OHM	6.42 ± 1.15	nd
M2-1 (*Scenedesmus obliquus*) ^1^	Aquaculture biofilters	OHM	149.00 ± 46.60 ^e^	0.01 ± 0.00 ^b^
M2-6 (*S. obliquus*) ^1^		OHM	25.10 ± 6.26 ^f^	nd
M3-9 (*S. obliquus*) ^1^		OHM	15.60 ± 2.83 ^f^	nd
M4-3 (*S. obliquus*) ^1^		OHM	2.66 ± 0.47	1.86 ± 0.32
M4-5 (*S. obliquus*) ^1^		OHM	63.10 ± 4.39 ^a,b^	nd
M2-5 (*S. obliquus*) ^1^		OHM	21.40 ± 3.91 ^c,d^	nd
M2-18 (*S. obliquus*) ^1^		OHM	10.5 ± 2.54	nd
*Scenedesmus* *obliquus* B	Estarreja wetlands	OHM	4.35 ± 0.46	nd
*S.* *obliquus* F		OHM	6.04 ± 0.66	nd
*S.* *obliquus* G		OHM	3.34 ± 0.50	nd
*Desmodesmus* *pleiomorphus* A		OHM	1.54 ± 0.05	nd
*D.* *pleiomorphus* E		OHM	0.76 ± 0.25	nd
*D.* *pleiomorphus* H		OHM	2.92 ± 0.86	nd
*D.* *pleiomorphus* M		OHM	1.81 ± 0.52	nd
*Scenedesmus* *quadricauda*	CBSC	OHM	8.27 ± 1.91	nd
**Eustigmatophyceae**				
*Nannochloropsis* sp.	SERI: NANNO-1 (GBSTICHO)	ASW ^2^	7.79 ± 2.11	nd
**Prymnesiophyceae**				
*Pavlova* *lutheri*	IPIMAR: SMBA 60	ASW ^2^	8.13 ± 1.83	nd
**Rhodophyceae**				
*Porphyridium* *aerugineum*	ACOI 1332	BG11	4.76 ± 1.31	nd

^a–g^ Means within the same column, without a common superscript, are significantly different (*p* < 0.05). ^1^ Wild species, identified using morphological characteristics and partial sequencing of chloroplastidial 16S rDNA. ^2^ ASW medium without 0.04 g/L SiO_3_. nd (not detected).

### 2.2. Antioxidant Capacity as ABTS Scavenging

Selection of the most promising microorganism(s) was based on assays for their antioxidant activity via complementary approaches; this strategy was expected to effectively overcome the (obvious) limitations of each analytical method proposed when considered independently. The first assay measured protection against oxidative damage by cell extracts and clearly indicated that isolate M2-1 (*Scenedesmus obliquus*) performed the best. Hence, only this isolate was subjected to complementary antioxidant assays as discussed in the next subsections. The results of the ABTS free radical scavenging assays are listed in Table 1, Table 2, for the intra- and extracellular extracts of cyanobacteria and microalgae, respectively.

The ascorbic acid-equivalent concentration in intracellular extracts ranged from 1.84 ± 0.57 AAU (Antioxidant Activity Units) for *Nodularia harveyana* to 38.10 ± 6.03 AAU for *Gloeobacter violaceus*. For the extracellular extracts, the values obtained were considerably lower [9]—ranging from (3.56 ± 0.76) × 10^−5^ AAU for *Nostoc muscorum* to 0.07 ± 0.01 AAU for *G. violaceus*.

Regarding intracellular extracts of microalgae, the ascorbic acid-equivalent concentration ranged from 1.54 ± 0.05 AAU for *Desmodesmus pleiomorphus* A to 149.00 ± 46.60 AAU for M2-1 (*S. obliquus*).

The levels of antioxidant activity found in this initial screening revealed that the intracellular extracts of microalgae are typically stronger than those of cyanobacteria: this is notably the case for isolate M2-1 (*S. obliquus*), the level of which is almost four-fold the highest value obtained with *G. violaceus*.

### 2.3. Antioxidant Capacity as Deoxyribose Protection

The protection of DNA from oxidative damage was analytically determined via protection of microalgal and cyanobacterial intracellular extracts upon induced deoxyribose damage [10]. This test was applied only to the most promising strains as concluded from inspection of Table 1, Table 2.

Since no extract exhibited pro-oxidant effects, the antioxidant power correlated linearly (and positively) to ability to inhibit deoxyribose degradation. Hence, intracellular extracts of the most promising strains were able to protect deoxyribose and decreased significantly its extent of degradation, which is an indication of hydroxyl radical scavenger capacity (see Table 3). The strongest protection was once again associated with strain M2-1, so only this extract underwent further assaying.

**Table 3:** Total antioxidant capacity (average ± standard deviation) of the best (**a**) cyanobacterial and (**b**) microalgal intracellular extracts, expressed as protection of deoxyribose, in percent inhibition of degradation by induced oxidation.

a	
**Cyanobacterium Strains**	**Antioxidant Power**
*Gloeobacter* *violaceus*	113.68 ± 2.81
M2-7 (*Limnothrix* sp.)	9.32 ± 0.63
J52 (*Anabaena planctonica*)	51.45 ± 5.04
*Scytonema* *obscurum*	16.48 ± 0.65
**b**	
**Microalga Species**	**Antioxidant Power**
*Haematococcus* *pluvialis*	20.34 ± 0.79
M2-1 (*S. obliquus*)	477.91 ± 161.95
M2-6 (*S. obliquus*)	138.46 ± 30.86
M2-5 (*S. obliquus*)	122.92 ± 9.57
M3-9 (*S. obliquus*)	11.75 ± 2.60
M4-5 (*S. obliquus*)	121.85 ± 46.97

### 2.4. Antioxidant Capacity as DNA Protection

**Figure 1:**
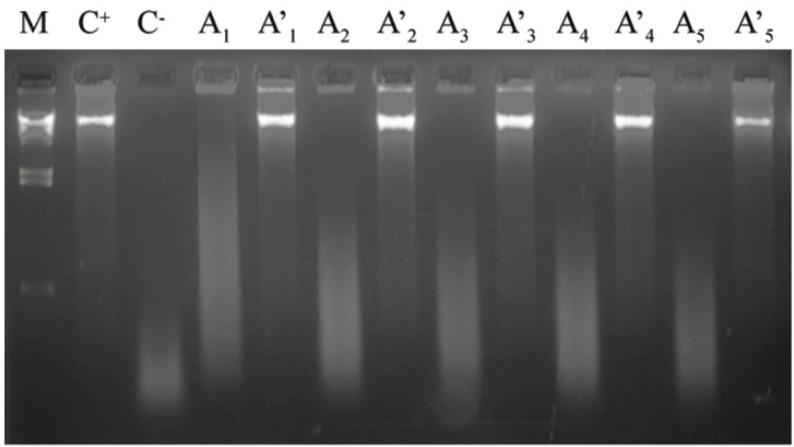
Agarose gel electrophoregram showing the damage induced to calf thimus DNA by Cu(II)-ascorbic acid, and the protective effect of various amounts of M2-1 extract: **M**—DNA molecular weight marker: λ DNA cut with *Hin*dIII; **C**^+^—1 μg of DNA; **C**^−^—1 μg of DNA plus Cu(II)-ascorbic acid; **A**—1 μg of DNA plus Cu(II)-ascorbic acid, and plus (**A**_1_) 200, (**A**_2_) 150, (**A**_3_) 100, (**A**_4_) 75 or (**A**_5_) 50 μL of extract; and A′—1 μg of DNA plus (**A′**_1_) 200, (**A′**_2_) 150, (**A′**_3_) 100, (**A′**_4_) 75 or (**A′**_5_) 50 μL of extract.

It is known that active oxygen species react with DNA (besides deoxyribose), and concomitantly produce strand breaks which can be detected using electrophoretic separation of the DNA fragments thus produced. Following Perez *et al.* [11], ascorbic acid and copper were utilized to induce oxidation (and consequent DNA cleavage); besides assessment of the antioxidant capacity of M2-1 (*S. obliquus*) extract. This method also addressed the question of whether a pro-oxidant effect was present (*i.e.*, if it could cause oxidation by itself). The results are depicted in Figure 1.

According to this method, the M2-1 extract protects calf-thymus DNA from induced oxidative damage. This effect became gradually more visible as the sample volume increased (see Figure 1, lanes A), and reached its maximum level when 200 μL of cell extract was incubated with DNA (see Figure 1, lane A1). Higher volumes (from 200 up to 400 μL) did not significantly affect the extent of protection (data not shown). In all cases, no significant pro-oxidant effect was detected (see Figure 1, lanes A′).

### 2.5. Antioxidant Capacity as Bacteriophage Protection

The use of bacteriophages as an *in vivo* system to assess antioxidant capacity is a relatively novel approach, yet results published elsewhere [12,13] unfolded its suitability as analytical method. In order to ascertain the antioxidant effect of M2-1 extracts, a preliminary assay used only ethanol/water (1:1, v/v) to determine whether ethanol would influence phage growth; it was concluded that it did not act as either oxidant or antioxidant (data not shown).

**Figure 2:**
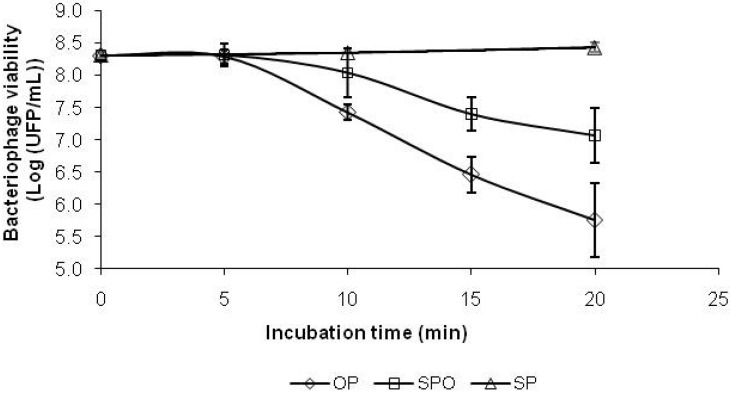
Evolution with time of the effect of M2-1 extract upon bacteriophage P22 viability (average ± standard deviation). The antioxidant effect was assessed as the difference between the observed infection of the bacterium by the phage in the presence of sample and oxidant (SPO), in the presence of oxidant only (OP) and in the presence of sample only (SP).

The antioxidant effect using this specific assay was calculated as the difference between the observed extent of infection of the bacterium by the virus in the presence of both M2-1 extract and H_2_O_2_ (SPO), and its counterpart in the presence of H_2_O_2_ only (OP). The inactivation curve of the P22 bacteriophage, which was attained by adding 250 mM H_2_O_2_, clearly unfolded a reduction in the number of phages available to infect *Salmonella* during the course of the experiment (see OP in Figure 2). By 20 min, a three log-cycle reduction had already been achieved relative to the initial phage numbers; this reduction was used as base line to assess the antioxidant activity of the M2-1 extract. 

Upon adding 50 mg·mL^−1^ of the said extract, the oxidant effect of H_2_O_2_ upon the phage was significantly reduced, *p* > 0.05 (see SPO in Figure 2). On the other hand, the M2-1 extract itself did not produce any damage to the phage (see SP in Figure 2). Furthermore, an important protective effect was conveyed by M2-1 extracts as soon as after 10 min of contact, which reached 1.5 log units by 20 min—although an essentially nil effect was observed during the first 5 min.

The M2-1 extract itself did not produce any damage to the virus, nor did the ethanol used as solvent interfere with the assay. Phage inactivation by externally induced chemical oxidation can accurately ascertain the effects of oxidative stress upon the biological integrity of DNA; alternatively, it may reflect damage to the proteinaceous phage capsid that hampers delivery of the inner DNA material. Our results showed that M2-1 extracts can effectively protect phage P22 against oxidative damage by H_2_O_2_, e.g., at 50 mg·mL^−1^.

### 2.6. Antioxidant Compounds

With the aim of identifying specific compounds that may account for the antioxidant capacity of M2-1 extract, several analyses were performed by HPLC; standards obtained either from commercial sources (e.g., lutein), or extracts of nettles and yellow pepper (e.g., zeaxanthin, cryptoxanthin, echinenone, neoxanthin, violaxanthin and luteoxanthin) were accordingly employed as reference. The retention times of these standards are depicted in Table 4. Lutein was quantified using a linear response factor, calculated from reference solutions (characterized by a correlation factor of 0.9976), whereas β-carotene, violaxanthin and neoxanthin concentrations were expressed as lutein-equivalents.

**Table 4:** Identification and quantification (average ± standard deviation) of antioxidant compounds in M2-1 extract, expressed as mg_equivalent__lutein_/g_microalga_.

Compound	Elution Time (min)	Antioxidant Concentration (mg_equivalent lutein_/g_microalga_)
Neoxanthin	5.9	0.56 ± 0.02
Violaxanthin	6.5	0.14 ± 0.01
Lutein	14.6	2.69 ± 0.09
Zeaxanthin	15.2	nd
β-Apo-8′-carotenal	20.1	(internal standard)
β-Carotene	34.5	0.40 ± 0.03

nd (not detected).

A particularly high lutein content (*i.e.*, 2.69 ± 0.09 mg·g^−1^) was recorded in the M2-1 extract, which suggests that the high antioxidant capacity observed is likely associated therewith. However, presence of β-carotene (0.40 ± 0.03 mg·g^−1^) and neoxanthin (0.56 ± 0.02 mg·g^−1^) cannot be ignored in this regard—including a putative synergistic effect with lutein [14].

### 2.7. Mutagenicity

**Figure 3:**
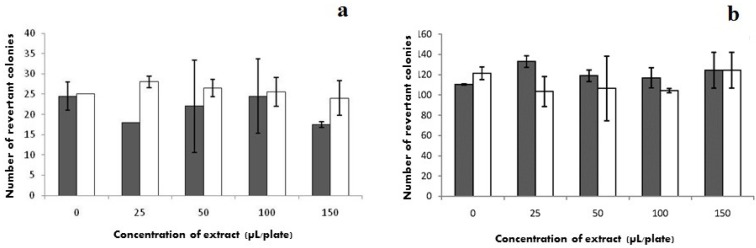
Variation with M2-1 extract concentration of the number of revertant colonies (average ± standard deviation) obtained with (**a**) TA 98 or (**b**) TA 100 strains, (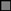) without S9 and (□) with S9.

The Ames test has been classically used to assess the mutagenic effects of various compounds, in either pure or crude forms. The results of this test on M2-1 extract, using the aforementioned TA98 and TA100 strains, are plotted in Figure 3a,b, respectively; the said extract did not lead to evidence of any direct mutagenic effect, even at the highest concentrations tested, since the number of revertants was statistically identical to that of spontaneous ones. Note that the Ames reversion assay was carried out on the sample solutions with TA98 and TA100, with and without the S9 (liver microssomal fraction) mixture [15].

In this test, histidine-requiring strains were used—each one having a different type of mutation on the histidine operon, besides other mutations that greatly increase their ability to detect chemical mutagens: e.g., strain TA 98 detects various frameshift mutagens, whereas strain TA 100 detects mutagens that cause base-pair substitutions [16]. The M2-1 extract did not produce evidence of any direct mutagenic effect, even at the highest concentration tested, because the number of revertant bacteria was statistically identical (*p* > 0.05) to that of the control.

## 3. Experimental Section

### 3.1. DNA Extraction and Agarose Gel Electrophoresis

Genomic cyanobacterial DNA and total microalgal DNA were isolated using the phenol/chloroform method, followed by ethanol precipitation [17,18]. Agarose gel electrophoresis was performed following standard protocols, using 1X TAE buffer [19]. DNA bands were visualized with ethidium bromide, under UV light. 

### 3.2. DNA Amplification, Purification, Cloning and Sequencing

The oligonucleotide primers used to amplify both the cyanobacterial 16S rDNA and the microalgal chloroplast 16S rDNA were CYA106F, CYA359F and CYA781R [18]. In addition, the universal bacterial primer 1541R (5′ AAGGAGGTGATCCAGCC 3′) was employed to obtain longer sequences of the cyanobacterial 16S rRNA genes.

The Polymerase Chain Reaction (PCR) assays were performed as described elsewhere [17], using the following profile: 40 cycles of 94 °C for 1 min, 50 °C for 1 min and 72 °C for 1 min, followed by an extension at 72 °C for 7 min. Visualization of the amplified DNA products was performed using 1.5% agarose gel electrophoresis. DNA fragments were isolated from agarose gels using the GFX PCR-DNA and Gel Band Purification kit (GE Healthcare, UK), according to the manufacturer’s instructions. The purified PCR products were cloned into the pGEM^®^-T Easy vector (Promega Corporation, Madison, WI, USA), and further used to transform *E. coli* DH5α competent cells—following again the manufacturer’s instructions. Colonies were screened for presence of the insert via colony-PCR, and subsequently grown overnight in liquid LB medium at 37 °C with shaking. Plasmid DNA was isolated using the GenEluteTM Plasmid Miniprep Kit (Sigma-Aldrich, St. Louis, MO, USA), and sequencing was performed at STAB Vida (Lisbon, Portugal). Computer-assisted sequence analysis and comparisons were performed using Vector NTI Advance 10 (Invitrogen Corporation, Carlsbad, CA, USA). Novel sequences associated with this study are available from GenBank, under the accession numbers depicted in Table 5.

**Table 5:** List of GenBank accession numbers of novel 16S rDNA sequences obtained, and closest cultured relatives.

Isolate	GenBank Accession Number	Closest Cultured Relative (% similarity, accession number) ^a^
J52	EU073188	*Anabaena* *planctonica* strain 71 (99%, AJ293108)
M2-7	EF634458	*Limnothrix* sp. strain CENA110 (99%, EF088338)
M2-1	EU073189	*Scenedesmus* *obliquus* strain UTEX 393 (98%, DQ396875)
M2-6	EU073191	*S.* *obliquus* strain UTEX 393 (92%, DQ396875)
M3-9	EU073193	*S.* *obliquus* strain UTEX 393 (92%, DQ396875)
M4-3	EU073194	*S.* *obliquus* strain UTEX 393 (92%, DQ396875)
M4-5	EU073195	*S.* *obliquus* strain UTEX 393 (92%, DQ396875)
M2-5	EU073190	*S.* *obliquus* strain UTEX 393 (99%, DQ396875)
M2-18	EU073192	*S.* *obliquus* strain UTEX 393 (99%, DQ396875)

^a^ Levels of similarity determined by BLAST.

### 3.3. Growth Conditions and Microorganisms

Environmental isolates and culture collection species/strains tested in this study are listed in Table 1 for cyanobacteria, and in Table 2 for microalgae.

Batch cultures were grown at 25 °C, in 200 mL of medium, under continuous illumination with fluorescent daylight (35 μmol_photon_ m^−2^ s^−1^), using preferentially OHM [20] and BG11/BG11_0_ [21] media, for microalgae and cyanobacteria, respectively. For marine isolates, ASW [22] and ASW-BG11 [23] media were used instead.

Genomic cyanobacterial DNA and total microalgal DNA were extracted according to Tamagnini *et al.* [17] and Burja *et al.* [18]. DNA amplification, purification, cloning and sequencing was performed at STAB Vida (Lisbon, Portugal); computer-assisted sequence analysis and comparisons were performed using Vector NTI Advance 10 (Invitrogen Corporation, Carlsbad, CA, USA). Novel sequences associated with this study were made available from GenBank.

### 3.4. Extracellular and Intracellular Extraction

Extracellular and intracellular extracts were collected according to Guedes *et al.* [24]. In short, 5 mL of a 30 day-old culture was centrifuged at 4000 rpm, for 7 min at 15 °C, and the supernatant (*i.e.*, extracellular extract) was collected. The pellet was then resuspended and homogenized in 5 mL of a mixture of ethanol and water (1:1 v/v); the cells were crushed in an Ultra Turrax T 18 homogenizer (Ika, Wilmington, NC, USA), at 14,000 rpm for 30 s, and then subjected to centrifugation at 4000 rpm, for 5 min at 15 °C; finally, the supernatant (*i.e.*, the intracellular extract) was collected.

### 3.5. Chlorophyll *a* Content

The total chlorophyll *a* content was determined after extraction of the cyanobacterial cells using 90%(v/v) methanol; absorbance was measured at 663 nm, and the equation μg_chlorophyll *a*_ mL^−1^ = 12.7 × Abs_663nm_—previously proposed by Meeks and Castenholz [25], was taken advantage of for quantification as reference basis.

Although a per biomass weight basis would in principle have been preferable because secondary rather than primary production was at stake, parallel determination of the content of chlorophyll *a* per dry biomass of a few species taken at random from each genus would not indeed alter the decision on selection of strains throughout screening, nor would it reverse any major conclusion; this is so because there is a strong correlation between dry weight and chlorophyll *a* content. The basis chosen is not only easier to quantitate, but also allows more meaningful discussion *vis a vis* with available data in the literature (owing to it being a regular trophic indicator). The known dependence of chlorophyll *a* levels on the physiological status of the cells did not apparently interfere with the figures generated, as in our case the cultures were all in their early stationary phase when harvested. 

### 3.6. ABTS Scavenging Assay

The radical-scavenging capacities of both extra- and intra-cellular extracts of microalgae and cyanobacteria were evaluated via the ABTS radical cation (ABTS^•+^) assay, as originally detailed by Re *et al.* [26] and recently refined by Gião *et al.* [27]—using, in the case of extracellular extracts, plain growth medium as control. The cation ABTS^•+^ was diluted with ultra-pure water, for extracellular extracts; and in a mixture of ethanol and water (1:1 v/v), for intracellular extracts; hence full quantification of both water- and lipid-soluble antioxidants was possible. The results were expressed in Antioxidant Activity Units (AAU), where 1 AAU is defined as 1 mg L^−1^ of equivalent ascorbic acid per μg of chlorophyll *a*.

### 3.7. Deoxyribose Protection Assay

The deoxyribose protection was quantified via a method described in detail elsewhere [28,29]. In brief, a 200 μL-sample of the extract of interest was added to 10 μL of 100 mmol L^−1^ deoxyribose, and incubated for 1 h at 37 °C—in the presence of 10 μL of 10 mmol L^−1^ Fe^3+^, 10 μL of 1 mmol L^−1^ 33% (w/v) H_2_O_2_, and 10 μL of 10 mmol L^−1^ EDTA, in a 24 mmol L^−1^ sodium phosphate buffer (pH 7.4) containing 15 mmol L^−1^ NaCl, to generate hydroxyl radicals.

The aforementioned radicals break deoxyribose into fragments which, in the presence of 1 mL of 1% (v/v) TBA in 0.05 mol L^−1^ NaOH, under acidic conditions (*i.e.*, 1.5 mL of 28% (v/v) tricloroacetic acid) and high temperature (*i.e.*, 100 °C for 15 min), give rise to a chromophore (malonaldehyde); this adduct was quantified by absorbance at 532 nm, using a Heλios α spectrophotometer (Unicam, Cambridge, UK). When antioxidants are present, they compete for hydroxyl radicals, thus decreasing the extent of fragmentation of deoxyribose.

All measurements were made against adequate blanks; and triplicate samples were taken, with analyses run in quadruplicate for each one.

### 3.8. DNA Protection Assay

Cell-free microalga extracts were prepared using 400 mg of lyophilized culture, resuspended in 12 mL of ethanol/water (1:1, v/v) and disrupted by ultrasonication for 15 min. Their protective effects against DNA oxidative damage induced by Cu(II)-ascorbic acid were assessed following Muñiz *et al.* [30] and Perez *et al.* [11]. For that purpose, each reaction mixture was prepared so as to contain 50 μg of calf thymus DNA, 10 mM of ascorbic acid and 100 μM of Cu(II), as well as various volumes of cell-free extract (*i.e.*, 50, 75, 100, 150 and 200 μL); the final volume was in all cases adjusted to 1 mL with 100 mM sodium phosphate buffer (pH 7.4).

The reaction mixtures were incubated in a shaking water bath, at 37 °C for 1 h, and 20 μL of each was loaded onto a 1.1% (w/v) agarose gel. Electrophoresis was performed according to the protocol described by Sambrook and Russell [19], using 1× TAE buffer; and DNA was visualized using ethidium bromide, under UV light.

### 3.9. Bacteriophage Protection Assay

Cell-free microalga extracts were prepared using 250 mg of lyophilized culture, resuspended in 5 mL of a mixture of ethanol/water (1:1, v/v), followed by cell disruption via sonication for 15 min, and final stirring for 10 min; the sample was then filtered through a sterile filter (0.22 μm). Details of this method were described previously [12,13].

The rationale for this methodology is a virucidal attack on *Salmonella* Typhimurium cells; when a challenging oxidant (250 mM H_2_O_2_) is initially applied, the bacteriophage infection capacity decreases; however, if protection occurs in the presence of antioxidant compound(s), the virus will recover its normal infection capacity.

More specifically, the stocks of phage P22 and *Salmonella* Typhimurium (ATCC 19585 P1) were prepared according to ATCC indications. Dilutions of the virus up to 10–12 were performed (in duplicate) in tryptone soy broth (TSB, from LAB M); 100 μL of each dilution was then mixed with 300 μL of *Salmonella* culture, harvested in the exponential phase—*i.e.*, a 1% (v/v) inoculum of an overnight culture in TSB at 37 °C, into fresh TSB was incubated at 37 °C for a further 2.5 h. Infection was allowed to proceed for 10 min, in a water bath kept at 37 °C; then, 100 μL was spread onto pre-dried plates of tryptone soy agar (TSA, from Biokar Diagnostic), and incubated at 37 °C for 24 h. Following incubation, the plaques were counted and the total viable phage numbers were accordingly calculated (and expressed in plaque-forming units per unit volume, PFU mL^−1^); the higher this figure, the stronger the infection capacity of the virus. Each mixture was left to react for 20 min, at room temperature (in duplicate), and was then quenched via addition of 50 μL of 500 U mL^−1^ catalase (Sigma). Aliquots of 100 μL were collected every 5 min for a period of 20 min, and serial decimal dilutions were performed up to 10^−6^.

For the infection stage, 100 μL of each phage dilution was added at a time to 300 μL of the suspension of *Salmonella* in the exponential phase (as described above), and incubated at 37 °C for 10 min. Then, 100 μL was spread onto pre-dried TSA plates (in duplicate), and incubated at 37 °C for 24 h; the viable phage number was again expressed as PFU mL^−1^. The difference between the viable phage numbers in the presence of sample and oxidant (SOP), and only in the presence of oxidant (OP), were considered as a datum point; when these were positive, then samples exhibited an antioxidant effect, and negative values indicated obviously otherwise.

### 3.10. Antioxidant Identification

Microalgal cell-free extracts were prepared using 40 mg of lyophilized culture, resuspended in 5 mL of acetone/hexane (1:1, v/v), and disrupted via ultrasonication for 15 min. Full description of the HPLC procedure is available elsewhere [24,31]; neoxanthin, violaxanthin, lutein, zeaxanthin, and β-carotene were assayed for, owing to their known antioxidant features.

Although the type of compounds extracted depends in general on the nature of the extracting solvent(s), water was not allowed in the aforementioned HPLC protocol; on the other hand, previous experience indicated that the compounds most likely to account for the antioxidant activity (*i.e.*, carotenoids) are quantitatively extracted irrespective of whether ethanol/water (1:1, v/v) or acetone/hexane (1:1, v/v) are employed.

### 3.11. Mutagenicity Assessment

An Ames reversion assay was carried out, in duplicate, on the M2-1 extract (25, 50, 100 and 150 μL/plate) using two types of reference strains, with and without the S9 (liver microssomal fraction) mixture [15]. Cell-free microalga extracts were accordingly prepared as described for the bacteriophage protection assay. The tester bacteria, *Salmonella* Typhimurium strains TA 98 and TA 100, were characterized elsewhere [15]. Reversion of specificity and activity of the S9 mix was confirmed with 5 μg of benzo-[*a*]-pyrene per plate. Quercetin (1 μg/plate) was used as positive control, and plain solvent as negative one.

### 3.12. Statistical Analyses

The experimental data did not typically follow a normal distribution, so non-parametric tests were applied to ascertain whether the type of microalga is a statistically significant parameter upon antioxidant activity, in its various forms. The tests were performed using SPSS, version 16.0.0 software [32].

## 4. Conclusions

The microalga M2-1 (*S. obliquus*) exhibits a particularly high antioxidant activity, among 23 microalgae and 18 cyanobacteria screened for that purpose. This antioxidant activity—assessed by such alternative methods as scavenging of free radicals, chemical protection of deoxyribose and DNA, and biological protection of bacteriophage upon *Salmonella* infection—seems to be associated chiefly with lutein. In addition, the antioxidant-rich extract of the M2-1 strain does not entertain any mutagenic effect, as assessed with specific *Salmonella* strains.

Therefore, strain M2-1 appears to be a particularly promising source of antioxidant additives for food formulation, or active principles for therapeutic use. Although low-cost cultivation media were utilized and no major constraints existed regarding growth, a careful economic analysis is still required prior to drawing any definite conclusions on industrial feasibility.
